# Description of a Malformation of the Duodenum

**Published:** 1845-07

**Authors:** Robert Boyd


*Description of a malformation of the duodenum.* By Robert Boyd, M. D. [Communicated by Robert Lee, M. D. F. R. S.] This morbid specimen was from a male still-born infant. The duodenum was enlarged, and appeared like a bladder two-thirds filled, and contained a greenish-coloured fluid. The lower or most distant part from the stomach was imperforate, and of larger calibre than the upper part, so that the malformed duodenum, as exhibited in the preparation, is somewhat of an oval shape. The intestine is completely closed by a transverse membrane at the lowest part; $2\frac{1}{4}$ inches above this, a valve extends across, nearly half closing the gut, proceeding from its concave side. Around the membrane which closes the duodenum the small intestine is attached, and, when dried and distended with air, is only about the thickness of a writing-pen. The author, in conclusion, refers to various writers who have given an account of similar divisions, and other malformations of the intestinal canal.—*Ibid.*

